# Altered brain activity during active forgetting in highly superior autobiographical memory: Evidence from an item-method directed forgetting

**DOI:** 10.1016/j.isci.2025.112607

**Published:** 2025-05-08

**Authors:** Valerio Santangelo, Tiziana Pedale, Sarah Daviddi, Ilenia Salsano, Simone Macrì, Patrizia Campolongo

**Affiliations:** 1 Department of Philosophy, Social Sciences & Education, University of Perugia, Piazza G. Ermini 1, 06123 Perugia, Italy; 2 Functional Neuroimaging Laboratory, Fondazione Santa Lucia IRCCS, Via Ardeatina 306, 00179 Rome, Italy; 3 Institute for Human Neuroscience, Boys Town National Research Hospital, 14090 Mother Teresa Lane, Boys Town, NE 68010, USA; 4 Centre for Behavioural Sciences and Mental Health, Istituto Superiore di Sanità, Viale Regina Elena 299, 00161 Rome, Italy; 5 Department of Physiology and Pharmacology “Vittorio Erspamer”, Sapienza University of Rome, Piazzale Aldo Moro 5, 00185 Rome, Italy; 6 CERC, Fondazione Santa Lucia IRCCS, Via del Fosso di Fiorano 64, 00143 Rome, Italy

**Keywords:** Medical imaging, Neuroscience

## Abstract

Individuals with highly superior autobiographical memory (HSAM) challenge current memory knowledge, yet it remains unclear if their superior memory stems from impaired forgetting. Using a directed forgetting paradigm, we examined this in 12 individuals with HSAM and 30 controls. During fMRI, participants viewed single words followed by “remember” or “forget” instructions. Five minutes later, participants performed a memory recognition task with old (previously studied) and new words. Behaviorally, both groups showed similar forgetting effects, recognizing fewer to-be-forgotten than to-be-remembered words. However, at the neural level, HSAM individuals showed increased activity in the dorsal and ventral frontoparietal regions during initial word presentation, prior to memory instructions. During active forgetting, they also showed increased activity in the anterior and posterior midline regions. These findings suggest that HSAM individuals require additional neural resources for active forgetting to compensate for their enhanced initial processing of stimuli, enabling them to match the forgetting performance of controls.

## Highlights

• Individuals with HSAM show forgetting performance comparable to controls
• HSAM enhances dorsal and ventral frontoparietal activity during stimulus processing
• Directed forgetting in HSAM enhances activity in anterior/posterior midline regions
• HSAM requires more neural resources to achieve typical forgetting performance

## Graphical abstract

**Figure:**
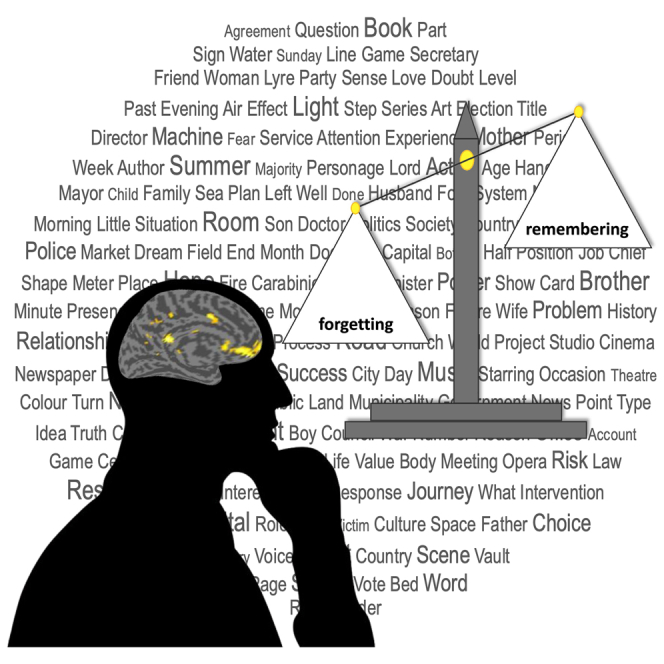

## Introduction

Forgetting has traditionally been viewed as a failure of memory associated with passive phenomena, such as the decay of old information or the interference of new material.^1^ This traditional view has been consistently challenged in recent decades, based on numerous findings highlighting “active” forgetting mechanisms (e.g., retrieval suppression), especially when considering unwanted or unneeded memories.^2^^,^^3^ Several lines of research are now converging to highlight an adaptive role for forgetting.^4^^,^^5^ This notion extends to the context of autobiographical memory (AM), which relies on the ability to recall episodic information from one’s own life. Typically, individuals retain in their AM meaningful events that are integrated into a coherent personal story,^6^^,^^7^ while discarding (i.e., forgetting) emotionally neutral and routine events that had no particular relevance to their lives.^8^ However, there are a small number of rare individuals – sometimes referred to by the media as “the people who never forget” – who can recall with a high degree of vividness and accuracy countless details of their lives that have very limited or no relevance, while typically showing average performance on standardized laboratory memory tests.^9^

The first modern evidence of this condition, called “highly superior autobiographical memory” (HSAM), was reported by Parker and colleagues.^10^ They described the case of Jill Price, who was able to reconstruct a personal experience from a given date with great accuracy. Since then, other groups of individuals with similar abilities have been discovered,^9^^,^^11^ and accumulating evidence has consistently shown enhanced mechanisms of AM retrieval in these individuals, who thus represent a model of enhanced memory capacity (see, Santangelo et al.^12^). The first functional magnetic resonance imaging (fMRI) study in individuals with HSAM showed enhanced neural activity during AM retrieval compared to controls, particularly during the initial phase related to AM access and construction.^11^ The increased neural activity included a circuit involving the ventromedial and dorsomedial prefrontal cortex (vmPFC/dmPFC) and the left temporo-parietal junction (TPJ). These regions also showed increased functional connectivity with a widespread network of regions during AM construction, including the hippocampus (from the vmPFC seed), medial and posterior parietal regions (from the dmPFC seed), and visual and auditory sensory cortices (from the TPJ seed). In a subsequent study, the vmPFC was found to be responsible for decoding AM temporal distance during AM memory in individuals with HSAM.^13^ The vmPFC was also found to support AM retrieval in a single case study of an elderly individual with HSAM.^14^ Other single case study findings highlighted the contribution of the precuneus^15^ and dorsolateral prefrontal cortex^16^ to AM retrieval in HSAM.

Although this previous literature has provided a first glimpse into how individuals with HSAM retrieve AMs, to date, there is no evidence regarding forgetting mechanisms in HSAM. In the current study, we therefore tested whether altered forgetting mechanisms might contribute to HSAM. We used an item-method directed forgetting paradigm, in which participants are instructed to “remember” or “forget” presented items, i.e., words.^17^^,^^18^ The rationale for using a word-based memory task involving episodic memory was to ensure that both individuals with HSAM and controls started from a comparable baseline in terms of memory performance. This was essential for isolating the specific mechanisms of forgetting without being influenced by the potential confound of heightened AM in the HSAM group. The item-method directed forgetting paradigm has been shown to effectively produce a recognition effect based on the directed instruction to remember or forget an item. This effect aligns with different theoretical accounts, such as the selective rehearsal account, which suggests that only to-be-remembered items are rehearsed, while to-be-forgotten items receive minimal rehearsal.^19^^,^^20^ However, an alternative inhibition account has gained prominence, proposing that to-be-forgotten items are actively suppressed following the forgetting instruction (see Anderson and Hanslmayr; Anderson and Levy^2^^,^^21^). This second account emphasizes the necessity of active and effortful cognitively demanding processes to achieve stimulus forgetting,^22^^,^^23^ ultimately leading to an impoverished memory representation for those stimuli that are still remembered.^24^ Moreover, this account is supported by findings showing that forgetting instructions elicit increased activity in the prefrontal cortex, suggesting a role in suppressing stimulus-related encoding^25^^,^^26^ (for a review, see Anderson and Hanslmayr^2^). The prefrontal cortex has also been found to suppress hippocampal-dependent encoding processes during intentional forgetting.^27^ This pattern of findings suggests a competitive dynamic between memory systems,^28^ with forgetting instructions reducing activity in episodic encoding systems (e.g., the hippocampus) while recruiting more generalized executive control regions (i.e., the prefrontal cortex).

At the same time, we investigated whether altered forgetting mechanisms are associated with enhanced stimulus processing in HSAM. Recently, we examined resting-state hippocampal functional connectivity in HSAM and controls.^29^ Individuals with HSAM showed reduced hippocampal connectivity with two large-scale brain networks involved in attentional orienting and saliency detection, namely the ventral fronto-parietal attentional network,^30^ including the temporo-parietal junction and inferior frontal gyrus, and the saliency network,^31^ including the anterior cingulate cortex and left and right insulae (for consistent findings, see Orwig et al.^32^). Conversely, individuals with HSAM showed increased hippocampal connectivity with visual and auditory sensory regions. This altered pattern of hippocampal connectivity may indicate that HSAM is associated with a reduced ability to discriminate and select salient information, with a subsequent increase in the likelihood of the “spontaneous” processing and elaboration of incoming sensory information, favoring stimulus encoding and consolidation regardless of its task-relevance.

**Figure 1:**
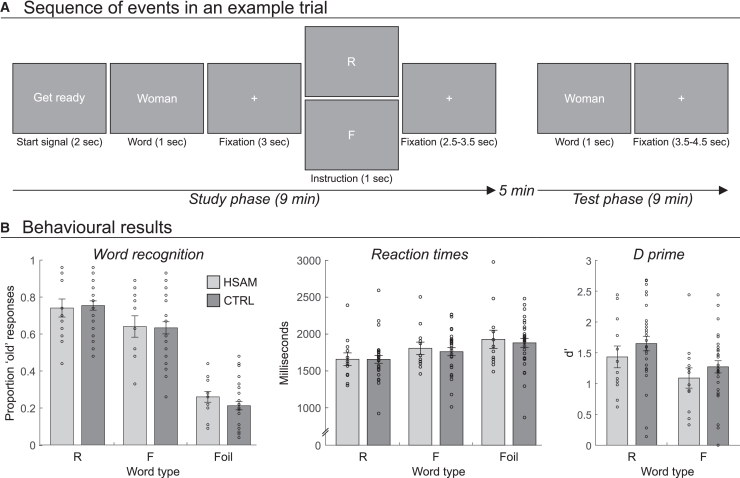
Task and behavioral results (A) Sequence of events in a sample trial, for both the study and test phases. The study phase consisted of the presentation of a start signal for 2 s, followed by the presentation of a word for 1 s; after a fixation cross (3 s of duration), a memory instruction was presented for 1 s, consisting of either the letter “R” or “F,” meaning “remember” or “forget” the previous word, respectively; after another fixation cross presented for a variable duration of 2.5–3.5 s, a new trial began. In the test phase, “old” or “new” words (i.e., words that were or were not presented in the study phase) were displayed for 1 s, followed by a fixation cross, with a variable duration of 3.5–4.5 s, in which the participants had to make an old/new judgment. (B) Proportions of “old” responses (left panel), reaction times (central panel), and d-prime score (right panel) in the word recognition task (test phase) as a function of the trial type (old trials: to-be-remembered, R, and to-be-forgotten, F, words; new trials: foil words) in the two groups. Bars in plots represent mean ± standard error of the mean; circles represent individual data points.

Accordingly, in the present study, we tested the hypothesis that HSAM might result from enhanced spontaneous processing of incoming stimuli, regardless of their relevance, accompanied by an altered mechanism of active forgetting, using an item-method directed forgetting. During functional magnetic resonance imaging (fMRI), we presented a series of words to a group of individuals with HSAM and a group of sex- and age-matched controls. Each trial began with the presentation of a word (i.e., the “stimulus presentation” period), followed by a memory instruction to either “remember” or “forget” the word (i.e., the “memory instruction” period). A few minutes after this study phase, participants completed an “old/new” memory recognition task in which they were presented with previously seen or completely new words (Figure 1A). If HSAM is based on increased stimulus processing independent of task relevance (in the current case, the instruction to remember a given stimulus/word), we would expect to find increased brain activity associated with word presentation in HSAM vs. controls. We would then expect that the instruction to actively forget this specific stimulus (i.e., the word) would reveal an altered pattern of neural activity in HSAM, consisting of an increased recruitment of neural resources to suppress spontaneous (and enhanced) word-related processing compared to controls. As predicted, along with similar behavioral outcomes between the two groups, we found increased neural activity during both initial stimulus processing and directed forgetting in HSAM individuals as compared to controls.

## Results

### Behavioral data

We computed the mean proportion of “old” responses made by participants in the test phase (i.e., in the memory recognition task) that were associated with the to-be-remembered (R), to-be-forgotten (F), or novel (Foil) words (Figure 1B, left panel). A two-way mixed analysis of variance (ANOVA) was performed on these data with the between-subjects factor of Group (HSAM vs. control) and the within-subjects factor of Word type (R, F, or Foil). The ANOVA revealed a main effect of Word type [F(2,80) = 169.882, *p* < 0.001, ƞ^2^ = 0.809]. Bonferroni-corrected post-hoc comparisons showed that participants made more recognition hits for R (0.75) than for F words (0.64; *p* < 0.001), revealing an active forgetting effect, while the proportion of recognition hits significantly decreased in the control condition, namely for Foil words (0.24; R vs. Foil: *p* < 0.001; F vs. Foil: *p* < 0.001). However, the active forgetting effect was similar in both groups, as indicated by the lack of the main effect of Group [F(1,40) = 0.130, *p* = 0.721, ƞ^2^ = 0.003] and the interaction between Group and Word type [F(2,22) = 0.568, *p* = 0.569, ƞ^2^ = 0.014]. A Bayesian ANOVA was performed to assess the strength of evidence for the absence of between-group differences.^33^ It provided substantial evidence (BF_01_ between 3 and 10) for both the lack of a group effect (BF_01_ = 3.82) and the lack of an interaction between group and word type (BF_01_ = 4.44).

For completeness, we also computed the mean reaction times associated with “old” responses as a function of the Word type (R, F, Foil), as well as the d’ (d-prime) associated with R and F trials, but failed to observe any between group difference. For the reaction times, a two-way mixed analysis of variance (ANOVA) was performed, including the between-subjects factor of Group (HSAM vs. Control) and the within-subjects factor of Word type (R, F, or Foil) (Figure 1B, central panel). This analysis revealed a consistent pattern of results with the proportion of “old” responses, i.e., a significant main effect of Word type [F(2,80) = 16.339, *p* < 0.001, ƞ^2^ = 0.290]. Post-hoc comparisons revealed faster reaction times for R (1664 ms) than for F (1790 ms; *p* < 0.001) words, with both conditions yielding faster reaction times than Foil words (1911 ms; R vs. Foil: *p* < 0.001; F vs. Foil: *p* < 0.001). Again, this pattern of results was not modulated by group: main effect of Group [F(1,40) = 0.110, *p* = 0.742, ƞ^2^ = 0.003] and Group × Word type interaction [F(2,80) = 0.183, *p* = 0.884, ƞ^2^ = 0.005]. The d' was calculated as follows: d' = z (hit rate) – z (false alarm rate) (Figure 1B, right panel). Separate d' scores were computed for each word type (R, F), and a two-way mixed ANOVA was performed with group (HSAM vs. Control) as the between-subjects factor and word type (R vs. F) as the within-subjects factor. The analysis revealed a significant main effect of word type [F(1,40) = 19.286, *p* < 0.001, ƞ^2^ = 0.325], indicating greater sensitivity for R (mean ± SD, 1.59 ± 0.64) words compared to F (1.22 ± 0.55) words. However, these effects were again not influenced by group, as neither the main effect of group [F(1,40) = 1.146, *p* = 0.291, ƞ^2^ = 0.028] nor the interaction group × word type [F(1,40) = 0.051, *p* = 0.823, ƞ^2^ = 0.001] reached significance.

### Functional magnetic resonance imaging data

**Figure 2:**
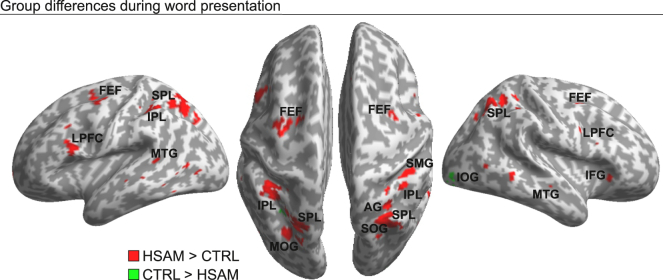
Brain activations during the word presentation period Brain regions showing the main effects of group (red map: HSAM > CTRL; green map: CTRL > HSAM) during word presentation superimposed on an inflated template (see also Table S1, supplemental information). Maps are displayed at a threshold of p-uncorrected = 0.001, with a minimum cluster size of 10 voxels.

The main aims of the present study were to examine whether individuals with HSAM recruit more neural resources than controls: 1) when processing any word regardless of the specific memory instruction (i.e., in the stimulus presentation period), and 2) during active forgetting in the memory instruction period (i.e., after the presentation of the “F” memory instruction). Regarding the first goal, the analysis (cf. GLM1, fMRI data analysis) revealed a different circuit of brain activity during word presentation in the HSAM vs. control group (Figure 2, red map; Table S1, supplemental information). This circuit involved several frontal and parietal regions. In the anterior brain, we found increased activity in the frontal eye-fields (FEF) and in the lateral prefrontal cortex (LPFC), bilaterally, as well as in the right inferior frontal gyrus (IFG). In the posterior brain, increased activity was found in the superior and inferior parietal lobule (SPL/IPL), bilaterally, and in the right angular and supramarginal gyri (AG and SMG), extending to the right superior occipital gyrus (SOG) and the left middle occipital gyrus (MOG), plus activity in the middle temporal gyrus (MTG), bilaterally. In contrast, increased activity during word presentation in the control vs. HSAM group was observed in only one region, the right inferior occipital gyrus (IOG; Figure 2, green map; Table S1, supplemental information).

**Figure 3:**
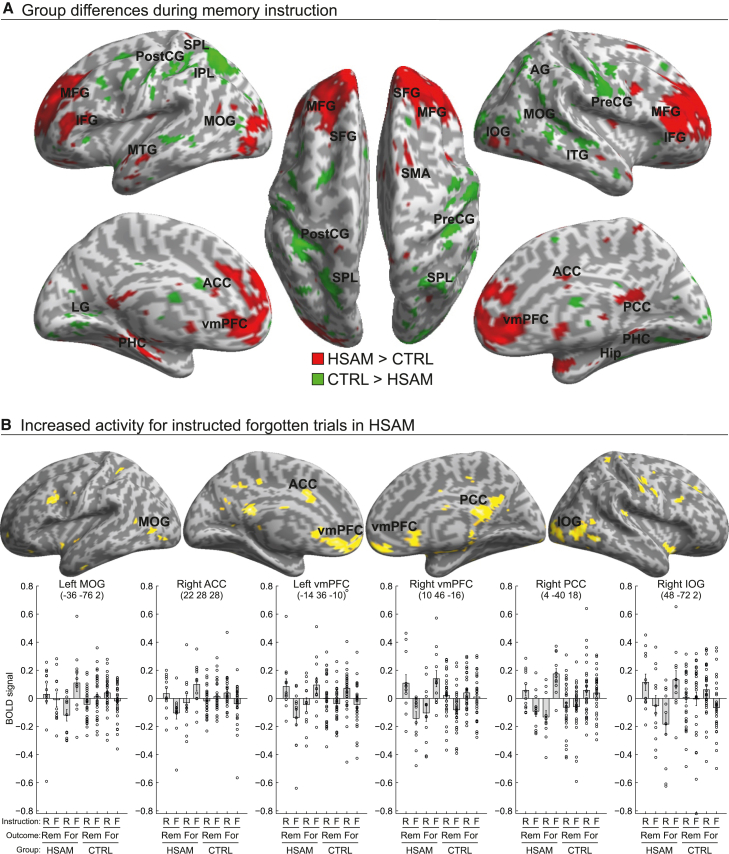
Brain activations during the memory instruction period (A) Brain regions showing the main effects of group (red map: HSAM > CTRL; green map: CTRL > HSAM) during memory instruction (i.e., directed encoding/forgetting) superimposed on an inflated template (see also Table S2, supplemental information). Maps are displayed at a threshold of p-uncorrected = 0.001, with a minimum cluster size of 10 voxels. (B) Brain regions showing increased activity in HSAM vs. control subjects during the presentation of the to-be-forgotten words that were actually forgotten in the test phase (see also Table S3, supplemental information), as revealed by the corresponding signal plots (compare the level of the BOLD signal for bar 4 vs. the other bars). For display purposes, maps are shown at a threshold of p-uncorrected = 0.005, with a minimum cluster size of 10 voxels. Bars in signal plots represent mean ± standard error of the mean; circles represent individual data points.

Regarding the second goal, the analysis (cf. GLM2, fMRI data analysis) revealed distinct circuits for the two groups in terms of active encoding/forgetting mechanisms. We found increased activity (Figure 3A, red map; Table S2, supplemental information) along the superior, middle, and inferior frontal gyri (SFG, MFG, and IFG), bilaterally, in HSAM compared to the control group. Medially, we found increased activation in the vmPFC and the anterior cingulate cortex (ACC), bilaterally, as well as in the right posterior cingulate cortex (PCC). Posteriorly, this circuit included the parahippocampal cortex (PHC), bilaterally, and the left MOG and right IOG, plus temporal activations (namely, in the left middle and superior temporal gyri, MTG/STG). Conversely, the main effect of control vs. HSAM subjects showed increased activity in the right precentral and left postcentral gyri (pre/postCG), in the right AG and the SPL, bilaterally, extending on the left hemisphere to the IPL and ventrally in the left lingual gyrus (LG) and calcarine cortex, and in the right hemisphere to the MOG and SOG. Activity also peaked in the left hippocampus, and laterally, in the right middle and inferior temporal gyri (MTG/ITG) (Figure 3A, green map; Table S2, supplemental information).

**Figure 4:**
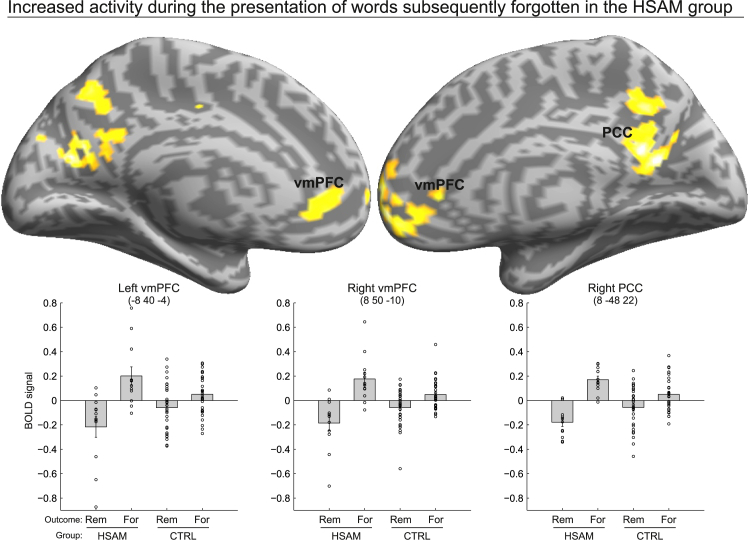
Brain activations for subsequently forgotten trials in HSAM Activity of the anterior and posterior midline regions during the presentation of subsequently forgotten words in the HSAM group (compare the level of BOLD signal for bar 2 vs. the other bars in the corresponding signal plots; see also Table S4, supplemental information) overlaid on an inflated template. Maps are displayed at a threshold of p-uncorrected = 0.001, with a minimum cluster size of 10 voxels. Bars in signal plots represent mean ± standard error of the mean; circles represent individual data points.

We then tested the contribution of these group-specific activations during active forgetting (i.e., the Memory instruction X Memory outcome × Group interaction). Six of the regions activated by the main effect of Group (HSAM > control) were found to selectively contribute to active forgetting in HSAM subjects, including the vmPFC, bilaterally, the right ACC, the left PCC, as well as the left MOG and the right IOG (Figure 3B; Table S3, supplemental information). As can be seen from the corresponding signal plots in Figure 3B, activity in these regions increased selectively for to-be-forgotten words (F) that were actually forgotten in the memory recognition task (For) in the HSAM group (compare bar 4 vs. the other bars). The same analyses that focused on the regions activated by the opposite main effect of Group (control > HSAM) did not reveal an analogous three-way interaction for the control group. Finally, three of the regions showing the three-way interaction in the HSAM group were found to overlap with the areas associated with the processing of words that were subsequently forgotten in the HSAM group, namely the left and right vmPFC, in the anterior midline, and the left PCC, in the posterior midline (Figure 4; Table S4, supplemental information). As shown by the corresponding signal plots in Figure 4, these regions showed increased activity in the HSAM group during the initial processing of words (i.e., during the stimulus presentation period) that were later not recognized as “old” during the memory recognition task.

## Discussion

The main aim of the present study was to investigate whether mechanisms of enhanced stimulus processing and altered active forgetting contribute to HSAM. We used an item-method directed forgetting task during fMRI, which allowed us to investigate the neural underpinnings of both mechanisms. Behaviorally, we found no difference between HSAM and control subjects in the magnitude of the active forgetting effect, i.e., the performance decrement in the memory recognition task when subjects were asked to forget vs. remember the stimulus. Both HSAM and control subjects recognized a comparable number of F vs. R words (10% fewer in HSAM and 12% fewer in controls), which was paralleled by a comparable increase in reaction times for F vs. R words (148 ms more in HSAM and 104 ms more in controls). Similarly, both groups showed similar rates of false alarms (i.e., foil words mistaken for “old” words; 26% and 21% for HSAM and control subjects, respectively), with similar reaction times (1935 and 1878 ms). This finding is very well in line with previous literature on HSAM, which has consistently found no differences between HSAM and control subjects on a variety of laboratory tests and cognitive functions, including verbal fluency, attention/inhibition, executive function, mnemonic discrimination, perception, working memory, emotional memory,^9^^,^^34^ and creativity.^35^ The present data extend this literature by demonstrating similar active forgetting effects in individuals with HSAM and related controls.

At the neural level, however, the scenario changes radically, revealing huge differences in the underlying neurobiological mechanisms recruited by HSAM subjects that may promote the achievement of comparable performance to controls during active forgetting. Group differences began to emerge from the stimulus presentation period, with HSAM subjects showing increased word-related activity well in advance of the memory instruction, i.e., before they knew whether the word was to be remembered or forgotten. Interestingly, we did not observe a differential contribution of the medial temporal lobe, traditionally considered a key region for episodic encoding,^36^^,^^37^^,^^38^^,^^39^ between the two groups. Conversely, most activations peaked along the prefrontal, lateral temporal, and parietal cortices. All of these regions have been typically implicated in episodic encoding in the literature. For instance, Grady and colleagues^40^ reported a study comparing the neural underpinnings of the episodic encoding of words vs. pictures, showing increased bilateral activity in the LPFC and temporo-parietal regions associated with language processing, including the MTG and lateral parietal cortices.^41^ Analogously, the activation of posterior parietal regions, including dorsal (the SPL) and ventral (the SMG and the AG in the IPL) areas, has been associated with episodic encoding, especially when testing for subsequent memory effects (i.e., when the encoding was successful^42^). Accordingly, Uncapher and Wagner^43^ reviewed several findings suggesting that encoding-related activity may be specifically associated with increased activation in the dorsal posterior parietal cortex. These regions are also known to be involved in goal-directed attention.^30^ Thus, our findings suggest an attentional influence on episodic encoding, increasing the likelihood that a given stimulus/event will be remembered later^44^^,^^45^ (for a meta-analysis, see Kim^46^). Ultimately, these activations may represent anticipatory encoding-related processes that begin in the HSAM group prior to the “active” encoding/forgetting period and independent of the memory instruction (i.e., to remember or forget the specific word stimulus).

However, alternative explanations for the current pattern of activations should be considered. Activity in the posterior parietal cortex has also been extensively implicated in episodic retrieval.^47^^,^^48^ For example, the “Attention to Memory” (AtoM) model^49^ proposes that dorsal posterior parietal cortex allocates attention during memory retrieval according to the subject’s goals (cf. top-down attentional control in perception), whereas ventral posterior parietal cortex would function by allocating attentional resources to salient memories/retrieval outputs (cf. bottom-up attention^30^). Moreover, other findings have shown that searching for information stored in memory engages lateral parts of the posterior parietal cortex, such as the AG^48^ and the lateral intraparietal sulcus.^50^ Accordingly, the currently observed extended activation, which includes both dorsal (the SPL, bilaterally), ventral (the IPL, bilaterally), and lateral (the right SMG and AG) posterior parietal activations, might be related to episodic retrieval. Speculatively, in this particular population of subjects, word presentation might act as a memory cue that triggers the retrieval of associated episodic memories,^9^^,^^51^ leading to increased activation in posterior parietal cortices. Finally, the circuit of regions showing increased activity during the stimulus presentation period in the HSAM group overlaps with regions associated with working memory maintenance. In a recent activation likelihood estimation (ALE) meta-analysis, Li and colleagues^52^ summarized 30 fMRI studies, reporting increased bilateral activity in the FEF, LPFC, IFG, and posterior parietal cortex during the maintenance period of a variety of visual working memory tasks. In principle, therefore, these activations could reflect an increased effort of working memory maintenance during the word presentation period in the HSAM groups. It should be noted, however, that this is unlikely given that the low level of working memory load, with only one word to maintain. Furthermore, previous studies have failed to find working memory differences in individuals with HSAM.^9^^,^^34^ Ultimately, although the current design did not allow us to disentangle between these potential accounts (namely, increased activity related to episodic encoding, episodic retrieval, or working memory maintenance), here we documented increased neural resources devoted to the processing of stimuli (i.e., words) not specifically related to autobiographical material (i.e., “laboratory” stimuli). Overall, this finding is consistent with the notion that HSAM may be sustained by enhanced and unconstrained spontaneous elaboration of information during encoding, irrespective of current goal-relevance, as postulated by Daviddi et al.^29^

The HSAM group also showed increased neural activations during the memory instruction period, specifically associated with to-be-forgotten words that they actually failed to remember during the memory recognition task. Over the past decades, the literature on active forgetting has highlighted several mechanisms associated with the suppression of unwanted memories, ranging from selective memory inhibition to more general suppression of the mnemonic process.^2^^,^^3^ However, all of these mechanisms seem to converge on the critical role of the prefrontal cortex in exerting top-down modulatory control over memory-related brain regions, such as the hippocampus. For example, Wylie and colleagues^26^ used an approach similar to the current study, asking participants to remember or forget single words. They reported increased activity in the right ventrolateral prefrontal cortex (specifically, the IFG) associated with words that were forgotten during the memory recognition task. Similarly, Oehrn and colleagues^27^ reported evidence from intracranial EEG recordings in pre-surgical epilepsy patients showing increased inhibition of hippocampal activity by the dorsolateral prefrontal cortex during voluntary forgetting with an item-method directed forgetting task. In contrast, our results did not show increased activations in the prefrontal-medial temporal brain circuitry associated with active forgetting in HSAM subjects, implying that these regions are not uniquely recruited irrespective of individual differences. Instead, what we found in HSAM subjects was increased activity in regions belonging to the anterior and posterior cortical midline (i.e., the vmPFC extending dorsally to the ACC and PCC, respectively) as well as occipital regions. Increased activity in the anterior and posterior midline regions, as part of the default mode network, has been shown to be positively correlated with episodic memory recall failure, whereas the deactivation of these regions predicts episodic memory success.^53^^,^^54^^,^^55^ Kim^46^ reported an ALE meta-analysis of 74 fMRI studies showing that activity in anterior and posterior midline regions, including the vmPFC – extending to the ACC – and the PCC, predicted subsequent episodic forgetting. Consistent with these findings, here we observed a selective increase in the anterior and posterior midline regions during the active forgetting phase in individuals with HSAM (vs. controls). Interestingly, the vmPFC and PCC were shown to overlap with the regions associated with subsequent memory recognition failure during the stimulus presentation period in the individuals with HSAM, i.e., when they were unable to recognize the “old” words. This suggests that these regions were already active during stimulus elaboration in the HSAM group, i.e., before the presentation of the remember/forget instruction, and were selectively associated with the words that were later forgotten in the memory recognition task.

While the vmPFC and PCC were found to increase their activity during both stimulus elaboration of subsequently forgotten words and active forgetting in the HSAM groups, the ACC in the dorsal midline was specifically recruited by HSAM individuals only during active forgetting of words. The ACC is a prefrontal region implicated in the cognitive control of demanding tasks.^56^ Increased ACC activity was found when participants were asked to suppress episodic memory retrieval, and the ACC has been shown to play a key role in this inhibitory process by modulating hippocampal activity through indirect pathways.^57^ This suggests that HSAM subjects may require increased activation in brain regions involved in top-down regulation in order to carry out the instruction to forget. In addition, there is evidence that the ACC is specifically involved in conflict monitoring: When a conflict is detected, the ACC is thought to send information to other brain regions in order to adjust behavior or cognitive response in accordance with goals and environmental demands.^58^^,^^59^ In light of this, HSAM subjects may show increased ACC activation during active word forgetting as a consequence of the fact that “forgetting” may be a highly conflicting demand for these individuals. This notion is consistent with the increased neural activity observed in the HSAM group during word presentation and with the hypothesis that these individuals have a tendency to encode stimuli regardless of their salience and task-relevance.^29^ Finally, individuals with HSAM showed increased activity in the visual cortex (namely, in left MOG and right IOG) during active forgetting. Given that these regions have previously been implicated in both the encoding^60^^,^^61^ and retrieval^46^^,^^62^ of visual information, their activation may indicate a different forgetting strategy adopted by individuals with HSAM, consisting of the reinstatement of alternative memories.^63^ Unfortunately, however, the current experimental design did not allow us to verify the specific strategy used by participants to suppress unwanted memories, which could be assessed in future research.

### Conclusions

In summary, despite similar behavioral outcomes associated with active forgetting in the two groups, we found that individuals with HSAM recruited dorsal and ventral frontoparietal regions compared to controls during initial stimulus processing (i.e., when the memory instruction to encode or forget the stimulus was not yet revealed). They then need to activate increased resources, mainly in anterior and posterior midline cortical regions, to achieve the same level of active forgetting performance as controls. We hypothesize that the increased neural activity during active forgetting might compensate for spontaneously enhanced stimulus processing in individuals with HSAM. Ultimately, the current findings extend the current knowledge of HSAM by revealing an altered neurobiological mechanism related to stimulus processing and active forgetting that may contribute significantly to the etiology of this rare memory condition.

### Limitations of the study

While we used a word-related memory task to assess forgetting, future research should consider using paradigms specifically designed to assess forgetting of autobiographical memories in individuals with HSAM. This would allow for a deeper understanding of the unique mechanisms underlying AM forgetting in this rare population.

## Resource availability

### Lead contact

Further information and requests for resources should be directed to and will be fulfilled by the lead contact, Valerio Santangelo (valerio.santangelo@unipg.it).

### Materials availability

This study did not generate new materials.

### Data and code availability

• The data reported in this study cannot be deposited in a public repository because the terms of our ethics approval do not allow public archiving of data, even in anonymised form, if any link to the individual’s identity cannot be excluded. Here, we report data from a rare population of individuals with HSAM who have released several interviewed to the national and international press. For this reason, the anonymization of the data cannot be fully guaranteed. Readers wishing to access the data should consult the lead contact. Access will be granted to named individuals in accordance with ethical procedures for the re-use of sensitive data. In particular, applicants will be required to complete a data sharing agreement.
• This article does not report original code.
• Any additional information required to reanalyze the data reported in this article is available from the lead contact upon request.

## Acknowledgments

Supported by the Italian Ministry of University and Research (J53D23017260001 to V.S. and P.C.) and the 10.13039/501100003196Italian Ministry of Health (RF-2019-12369567, to P.C. and V.S.).

## Author contributions

Conceptualization, V.S. and P.C.; methodology, V.S. and S.D.; investigation, T.P., S.D., and I.S.; formal analysis, V.S., T.P., and S.D.; data curation, V.S.; writing – original draft, V.S. and T.P.; writing – review and editing, all authors; funding acquisition, V.S. and P.C.

## Declaration of interests

The authors declare no competing interests.

## STAR★Methods

### Key resources table

**Table:** 

REAGENT or RESOURCE	SOURCE	IDENTIFIER
**Software and algorithms**		

MATLAB 7.5	The MathWorks Inc., Natick, MA	https://mathworks.com/products/matlab.html
Cogent2000 Toolbox	Wellcome Laboratory of Neurobiology, University College London	https://github.com/lnnrtwttkhn/Cogent2000
MATLAB R2018a	The MathWorks Inc., Natick, MA	https://mathworks.com/products/matlab.html
SPM12		https://www.fil.ion.ucl.ac.uk/spm/software/spm12/

### Experimental model and study participant details

#### Screening procedure and participants details

Since 2015, we have screened individuals with HSAM in the Italian population using the procedure previously reported by Santangelo et al.^11^ Briefly, this consisted of the administration of the “Public Events Quiz” and the “Random Dates Quiz”,^9^ adapted for the Italian population.^11^ The Public Events Quiz consisted of thirty questions based on public events selected from five categories: sporting events, political events, notable negative events, events related to famous people, and holidays. Fifteen of these questions asked participants to retrieve the specific date of a given significant public event, while the remaining fifteen questions asked participants to associate a given date with a famous significant public event. The random date quiz consisted of 10 computer-generated random dates ranging from the individual’s age of fifteen to the day before the test. Individuals were asked to provide three details for each date: (1) the day of the week; (2) a description of a verifiable event (i.e., one that could be confirmed using a search engine) that occurred within one month before and after the generated date; (3) a description of a personal autobiographical event.

Before the study began, we asked the HSAM individuals in our cohort if they were willing to participate in the current experiment. Twelve HSAM participants (9 males and 3 females; mean age: 35.3 years; range: 20–60 years) agreed to participate in the study. These individuals with HSAM averaged 55% accuracy on the public event quiz and 65% accuracy on the random date quiz, which is consistent with previous literature.^9^^,^^14^ We therefore examined whether this sample size (*N* = 12) was large enough to achieve statistical power, which was confirmed by a behavioral pilot study (see section below). Individuals with HSAM were compared with a group of 30 age- and sex-matched controls (21 males and 9 females; mean age: 35.7 years; range: 20–59 years) recruited through contacts in the community, none of whom reported HSAM or other superior memory abilities (for a similar approach, see LePort et al.^9^). All participants gave written consent to the study, which was approved by the independent Ethics Committee of the Fondazione Santa Lucia IRCCS.

#### Behavioral pilot study

We conducted a behavioral pilot study with 12 control subjects (8 females; 18–43 years; mean age = 24.67 years; SD = 6.5 years). Stimuli, design, and procedure were identical to those used in the main fMRI experiment. A within-subjects analysis of variance (ANOVA) was conducted to analyze the proportion of ‘old’ responses for the to-be-remembered, to-be-forgotten, and foil (i.e., new) words presented in the test phase. The ANOVA revealed a significant effect of word type [F(2,22) = 51.802, *p* < 0.001, ƞ^2^ = 0.825], indicating that participants gave more ’old’ responses for to-be-remembered (0.73) than for to-be-forgotten (0.53) words [t(11) = 3.139, *p* = 0.009, Cohen’s d = 0.906], which in turn were associated with higher than foil words (0.15) [t(11) = 7.087, *p* < 0.001, Cohen’s d = 2.046]. Collectively, the pilot study confirmed that a sample size of 12 subjects was large enough to obtain a statistically significant active forgetting effect (with large effect sizes: ƞ^2^ = 0.825 and Cohen’s d = 0.906), with participants’ recognition accuracy being higher for words to be remembered than for words to be forgotten.

### Method details

#### Stimuli and task

During the fMRI scan, participants were administered an adapted version of the direct forgetting task introduced by Wylie et al.^26^ This paradigm consisted of a study phase followed by a test phase (Figure 1A). Stimulus presentation and response collection was accomplished using the Cogent 2000 Toolbox (Wellcome Laboratory of Neurobiology, University College London) running on MATLAB 7.5 (The MathWorks Inc., Natick, MA). The experimental stimuli consisted of 200 Italian words selected from the CoLFIS database (https://www.istc.cnr.it/en/grouppage/colfis; for the full list of the words used in the experiment see Table S5, supplemental information). Specifically, the words consisted of nouns – uniquely specified, without the inclusion of synonyms or similar words – with a mean ± SD of 6.25 ± 1.77 letters and 2.68 ± 0.74 syllables.

In the study phase, participants were presented with a sequence of 54 words randomly selected from the 200-word database. Figure 1A shows an example trial that began with the presentation of a “get ready” signal for 2 s in the center of the screen. This was followed by the presentation of a word for 1 s. After a fixation cross was presented for 3 s, an instruction appeared for 1 s. The instruction consisted of either the letter “R”, i.e., to remember the word, or “F”, i.e., to forget the word. Participants were instructed to use whichever strategy they preferred to complete the task. After the presentation of a fixation cross (with a variable duration of 2.5–3.5 s, uniformly distributed), a new trial began. The study phase consisted of 54 trials (half R and half F trials, randomized) with a total duration of 9 min. Approximately 5 min later (i.e., after the administration of the anatomical scan, see magnetic resonance imaging section below), the test phase began (Figure 1A). On each trial, a word was presented for 1 s followed by a fixation cross, with a variable duration of 3.5–4.5 s, evenly distributed. Participants had to perform an “old/new” recognition memory task, i.e., they were instructed to judge whether the word was “old” (i.e., seen in the study phase, regardless of the memory instruction) or “new”. The test phase consisted of 108 trials: 54 old (27 R and 27 F) and 54 new (i.e., foil) words, randomized, for a total duration of 9 min.

Before the experiment began, participants were told that on each trial a word would be presented in the center of the screen and that after a short interval they would be asked to remember or forget the word. They were also told that a memory recognition test would follow, but were not told that they would be asked to recognize all the words seen in the study phase (i.e., both “R” and “F” words).

#### Magnetic resonance imaging

A Siemens Prisma (Siemens Medical Systems, Erlangen, Germany) operating at 3T and equipped for simultaneous multi-slice for echo-planar imaging (EPI) was used to acquire the functional and structural magnetic resonance images. A quadrature volume head coil was used for radio frequency transmission and reception. Head motion was minimized by mild restraint and cushioning. The experiment included 2 functional runs, one for the study and one for the test phase, plus a structural scan. Each functional run included 501 volumes. Sixty slices of functional MR images were acquired using blood oxygenation level-dependent imaging (3 × 3 mm, 2.4 mm thick, 50% distance factor, repetition time (TR) = 1.1 s, time echo (TE) = 30 ms), covering the entire cortex. For the structural scan, a T1-weighted structural scan (MPRAGE) was acquired (176 slices, 1 mm thick, TR = 2.5 ms, TE = 2 ms, TI = 210 ms, FOV = 240 × 256 mm^2^, matrix size = 240 × 256, flip angle = 8°).

### Quantification and statistical analysis

#### fMRI data analysis

We used SPM12 (Functional Imaging Laboratory, University College London) implemented in MATLAB R2018a (The MathWorks Inc., Natick, MA) for data pre-processing and statistical analyses. After having discarded the first four volumes, all images were (i) corrected for head movements, (ii) unwarped by using a field map, (iii) slice-time corrected using the first acquired slices as a reference (four slices acquired in parallel), (iv) co-registered to high-resolution structural data, and (v) normalized to the standard SPM12 EPI template, resampled to 2 mm isotropic voxel size. Finally, the functional images were smoothed with an 8-mm FWHM Gaussian kernel. Time series at each voxel for each participant were high-pass filtered at 128 s and pre-whitened through autoregressive model AR(1).

The primary aim of the present study was to determine whether individuals with HSAM recruit more neural resources than controls during word-related processing in the (i) word presentation period and during active forgetting in the (ii) memory instruction period. Accordingly, we constructed two different general linear models (GLMs) to account for the neural activity associated with word presentation before the memory instruction (GLM1) and with the presentation of the memory instruction (GLM2). For both GLMs, statistical inference was based on a random effects approach, which consisted of two steps: first-level multiple regression models estimating the contrasts of interest for each subject, followed by second-level analyses for statistical inference at the group level (with non-sphericity correction; Friston et al.^64^).

For GLM1 (“stimulus presentation period”), the first-level models had separate regressors related to the outcome of the test phase (remembered, “Rem”, vs. forgotten, “For”, words, i.e., correct vs. incorrect recognition as ‘old’ words, respectively). The two conditions were modeled as box-car functions, time-locked to the onset of the word, with a duration of 4 s (i.e., until the onset of the memory instruction; see Figure 1A, left panel). For each subject, we estimated contrast images for each condition, thus resulting in 2 contrast images per group: “processing of subsequently remembered words” (Rem) and “processing of subsequently forgotten words” (For). For the second-level group-analysis, the single-subject contrast images of the parameter estimates were entered into a 2 x 2 mixed design ANOVA with Group (HSAM vs. control) as the between-subjects factor and Outcome (Rem vs. For) as the within-subjects factor. Consistent with the current aims of the study, GLM1 was primarily used to search for differences in neural activity during word presentation between the two groups (i.e., the main effect of the group, see Figure 2; Table S1, supplemental information). The statistical threshold was set at p-family-wise error (FWE)-corrected <0.05 at the voxel level, with the whole brain considered as the volume of interest. GLM1 was also used to better characterize the word-related neural activity associated with subsequently forgotten words in the HSAM group (see the next paragraph).

For GLM2 (“memory instruction period”), the first-level models had separate regressors related to the memory instructions (R vs. F), split according to the outcome of the test phase (Rem vs. For words). The four conditions were modeled as box-car functions, time locked at the onset of the memory instruction, with a variable duration (range: 3.5–4.5 s, depending on the length of the fixation cross; see Figure 1A. For each subject, we estimated contrast images for each condition, resulting in 4 contrast images per group: “R_Rem”, “F_Rem”, “R_For”, and “F_For”. For the second-level group-analysis, the single-subject contrast images of the parameter estimates were entered into a 2 x 4 mixed design ANOVA with group (HSAM vs. control) as the between-subjects factor and Word type (R_Rem, F_Rem, R_For, and F_For) as the within-subjects factor. First, we highlighted the different brain activity in the two groups during active encoding/forgetting, i.e., the main effects of group. The statistical threshold was set at p-FWE-corrected <0.05 at the voxel level, considering the whole brain as the volume of interest. This comparison allowed us to highlight different circuits recruited by HSAM and control groups (Figure 3A; Table S2, supplemental information). The resulting activations were used to define regions of interest (ROIs) that were then used to test for condition-specific effects in interaction with the group variable (i.e., the three-way interaction). For this, we considered spheres (10-mm radius) centered on the regions activated by the main effects of group (i.e., HSAM > CTRL; and CTRL > HSAM; Table S2, supplemental information) as the volume of interest (using the small volume correction SPM function). This approach allowed us to selectively highlight the regions showing increased activity during active forgetting of words that were effectively forgotten in the memory recognition task (i.e., in the test phase) in the HSAM group. Finally, to further characterize the function of these regions, we examined whether they overlapped with regions associated – in the word presentation period – with subsequently forgotten words in the HSAM group. For this analysis, we also considered 10-mm spheres as well, now centered on the regions showing increased activity during active forgetting (i.e., the three-way interaction effect; Table S3, supplemental information), as the volume of interest (small volume correction). Within these ROIs, we searched for significant activity during the presentation of words that were subsequently forgotten (GLM1). As an additional constraint, we only considered voxels for this analysis that showed overall activation across all conditions and groups (T-contrast, P-uncorrected = 0.001; Figure S1, supplemental information), ensuring that we only selected regions involved in the stimulus presentation period (for a similar approach, see Büchel et al. and Santangelo et al.^65^^,^^66^).

## Footnotes

Supplemental information can be found online at https://doi.org/10.1016/j.celrep.2025.115725.
