# Supplementary material for: Altered brain activity during active forgetting in highly superior autobiographical memory: Evidence from an item-method directed forgetting
Source: iScience. 2025 May 8;28(6):112607. doi: 10.1016/j.isci.2025.112607 (PMC12150045; doi:10.1016/j.isci.2025.112607)
Supplement: Document S1. Figure S1 and Tables S1–S5 [file mmc1.pdf]

iScience, Volume *28*

Supplemental information

Altered brain activity during active forgetting in highly superior autobiographical memory: Evidence from an item-method directed forgetting

Valerio Santangelo, Tiziana Pedale, Sarah Daviddi, Ilenia Salsano, Simone Macrì, and Patrizia Campolongo

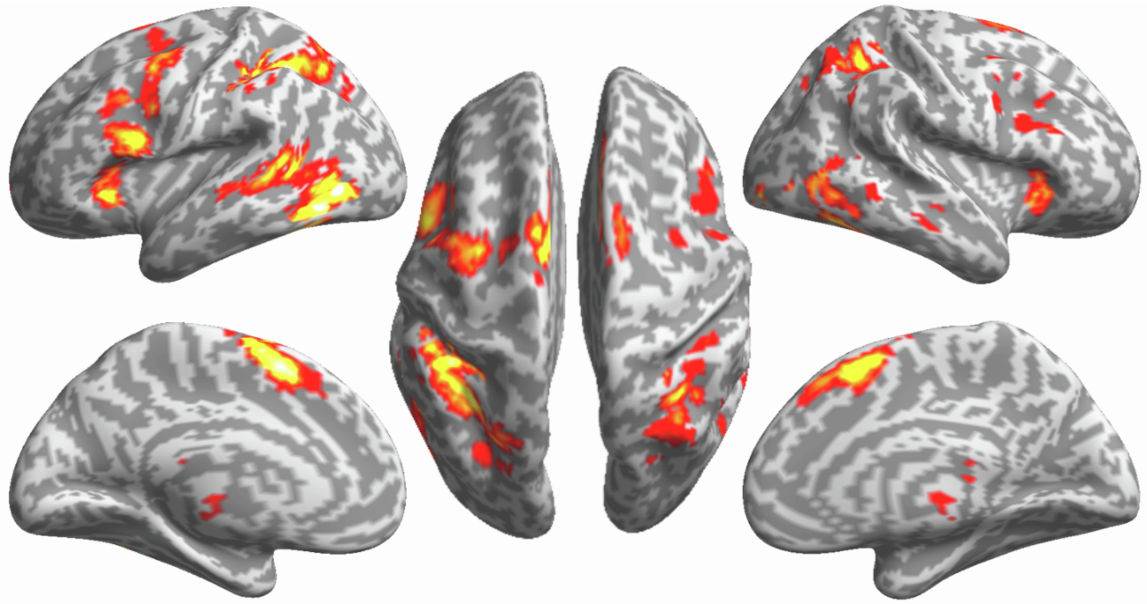

Figure S1. Overall activation during word presentation. Maps are displayed at a threshold at p-uncorrected = .001, with a minimum cluster size of 10 voxels.

Table S1. MNI coordinates (x, y, z), Z-values, and p-FWE-corrected values for areas showing a main effect of group, HSAM vs. CTRL group, and vice versa, during the stimulus presentation period.

	**x y z**	**Z-value**	**p-FWE-corr**
HSAM > CTRL			
Right IPL	26 -48 50	Inf.	< .001
Left SPL	-26 -56 54	7.61	< .001
Left OFC	-12 64 -6	7.29	< .001
Right AG	28 -62 50	6.16	< .001
Right SMG	42 -34 44	6.06	< .001
Right SPL	32 -66 58	5.95	< .001
Left FEF	-24 -4 44	5.91	< .001
Left LPFC	-56 14 32	5.86	< .001
Right FEF	32 0 44	5.63	.002
Right MTG	68 -14 -14	5.62	< .001
Left IPL	-40 -42 40	5.50	.001
Right SOG	34 -76 48	5.44	.001
Right LPFC	50 10 30	5.34	.001
Right IFG	38 30 -2	5.21	.003
Right Caudate	10 12 0	5.21	.003
Right SFG	12 28 48	5.14	.004
Left MOG	-28 -78 34	5.03	.006
Left MTG	-58 -56 10	4.90	.012
CTRL > HSAM			
Right IOG	38 -88 -6	Inf.	< .001

Note: IPL: inferior parietal lobule; SPL: superior parietal lobule; OFC: orbitofrontal cortex; AG: angular gyrus; SMG: supramarginal gyrus; FEF: frontal eye-fields; LPFC: lateral prefrontal cortex; MTG: middle temporal gyrus; SOG: superior occipital gyrus; IFG: inferior frontal gyrus; SFG: superior frontal gyrus; MOG: middle occipital gyrus; IOG: inferior occipital gyrus.

Table S2. MNI coordinates (x, y, z), Z-values, and p-FWE-corrected values for areas showing a main effect of group, HSAM vs. CTRL group, and vice versa, during the memory instruction period.

	**x y z**	**Z-value**	**p-FWE-corr**
HSAM > CTRL			
Left SFG	-16 58 10	Inf.	< .001
Right SFG	14 62 8	Inf.	< .001
Right vmPFC	14 40 -12	Inf.	< .001
Right IFG	42 34 8	Inf.	< .001
Right ACC	18 38 22	7.81	< .001
Right MFG	34 48 20	7.61	< .001
Left ACC	-16 34 28	7.52	< .001
Left MOG	-36 -84 -2	7.31	< .001
Left vmPFC	-14 46 -10	6.58	< .001
Right PHC	28 6 -30	6.46	<.001
Left Cerebellum	-46 -46 -42	6.24	< .001
Left PHC	-22 -24 -22	6.12	< .001
Right PCC	12 -44 20	5.31	.002
Left FG	-38 -56 -14	4.70	.024
Right SMA	14 20 64	4.69	.025
Right IOG	42 -76 -4	5.76	< .001
Right ITG	48 -60 -14	5.33	.001
Left Globus Pallidus	-14 2 -4	4.97	.007
Left STG	-48 -2 -6	4.69	.024
Left MTG	-40 -62 16	4.53	.047
CTRL > HSAM			
Left OFC	-12 64 -6	Inf.	< .001
Right MOG	32 -94 10	Inf.	< .001
Left SPL	-24 -68 48	7.13	< .001
Right AG	58 -60 32	6.52	< .001
Right SPL	34 -66 56	6.50	< .001
Left IPL	-40 -38 40	6.42	< .001
Left SFG	-6 66 22	5.66	< .001
Left Cerebellum	-22 -84 -24	5.53	< .001
Right Calcarine	28 -66 -4	5.36	.001
Right MTG	60 -58 18	5.35	.001
Right SMG	40 -32 42	5.25	.002

Right Cerebellum	6 -82 -34	5.23	.002
Left MOG	-48 -78 18	5.22	.002
Right LG	26 -74 -4	5.06	< .001
Right SOG	28 -82 42	5.03	.006
Left Calcarine	-12 -90 -8	5.03	.005
Right PreCG	52 -6 50	4.76	.019
Right Caudate	10 8 8	4.71	.023
Right Insula	34 18 -16	4.71	.023
Right PostCG	56 -12 34	4.70	.023
Right MFG	40 22 54	4.64	.030
Right IFG	38 30 -2	4.62	.030
Left Hippocampus	-32 -32 0	4.62	.033
Right ITG	60 -14 -28	4.52	.049

Note: SFG: superior frontal gyrus; vmPFC: ventromedial prefrontal cortex; IFG: inferior frontal gyrus; ACC: anterior cingulate cortex; MFG: middle frontal gyrus; MOG: middle occipital gyrus; PHC: parahippocampal cortex; PCC: posterior cingulated cortex; FG: fusiform gyrus; SMA: supplementary motor area; IOG: inferior occipital gyrus; ITG: inferior temporal gyrus; STG: superior temporal gyrus; MTG: middle temporal gyrus; OFC: orbitofrontal cortex; SPL: superior parietal lobule; AG: angular gyrus; IPL: inferior parietal lobule; SMG: supramarginal gyrus; LG: lingual gyrus; SOG: superior occipital gyrus; PreCG: precentral gyrus; PostCG: postcentral gyrus.

Table S3. MNI coordinates (x, y, z), Z-values, and p-FWE-corrected values for areas showing the three-way interaction between memory instruction (R vs. F), memory outcome (Rem vs. For), and group (HSAM vs. CTRL).

	**x y z**	**Z-value**	**p-FWE-corr**
Right vmPFC	10 46 -16	3.32	.017
Left vmPFC	-14 36 -10	3.33	.017
Right ACC	22 28 28	3.52	.009
Left MOG	-36 -76 2	3.20	.025
Right PCC	4 -40 18	3.60	.007
Right IOG	48 -72 2	3.57	.008

Note: vmPFC: ventromedial prefrontal cortex; ACC: anterior cingulate cortex; MOG: middle occipital gyrus; PCC: posterior cingulate cortex; IOG: inferior occipital gyrus.

Table S4. MNI coordinates (x, y, z), Z-values, and p-FWE-corrected values for those areas showing the three-way interaction (cf. Table S3) that overlapped with those areas showing increased activity during the presentation of subsequently forgotten words in the HSAM group.

	**x y z**	**Z-value**	**p-FWE-corr**
Right vmPFC	8 50 -10	3.50	.011
Left vmPFC	-8 40 -4	3.25	.024
Right PCC	8 -48 22	3.31	.020

Note: vmPFC: ventromedial prefrontal cortex; PCC: posterior cingulate cortex.

Table S5. List of the 200 Italian words used as stimuli in the active forgetting task.

Accordo	Domanda	Libro	Parte	Segno
Acqua	Domenica	Linea	Partita	Segretario
Amico	Donna	Lira	Partito	Senso
Amore	Dubbio	Livello	Passato	Sera
Aria	Effetto	Luce	Passo	Serie
Arte	Elezione	Macchina	Paura	Servizio
Attenzione	Esperienza	Madre	Periodo	Settimana
Autore	Estate	Maggioranza	Personaggio	Signore
Azione	Età	Mano	Pezzo	Sindaco
Bambino	Famiglia	Mare	Piano	Sinistra
Bene	Fatto	Marito	Piede	Sistema
Bisogno	Festa	Mattina	Poco	Situazione
Camera	Figlio	Medico	Politica	Società
Campagna	Film	Mercato	Polizia	Sogno
Campo	Fine	Mese	Porta	Sole
Capitale	Fondo	Metà	Posizione	Spazio
Capo	Forma	Metro	Posto	Speranza
Carabiniere	Forza	Ministro	Potere	Spettacolo
Carta	Fratello	Minuto	Presenza	Squadra
Casa	Fuoco	Modo	Presidente	Stagione
Caso	Futuro	Moglie	Problema	Storia
Centro	Gente	Momento	Processo	Strada
Chiesa	Gioco	Mondo	Progetto	Studio
Cinema	Giornale	Morte	Programma	Successo
Città	Giorno	Musica	Protagonista	Teatro
Colore	Giro	Natura	Prova	Tempo
Colpo	Giudice	Nome	Pubblico	Terra
Comune	Governo	Notizia	Punto	Tipo
Condizione	Gruppo	Notte	Ragazzo	Titolo
Consiglio	Guerra	Numero	Ragione	Ufficio
Conto	Idea	Occasione	Rapporto	Uomo
Controllo	Immagine	Occhio	Ricerca	Valore
Corpo	Incontro	Opera	Rischio	Verità
Corso	Interesse	Ora	Risposta	Viaggio
Cosa	Intervento	Ordine	Risultato	Vita

Crisi	Italiano	Ospedale	Ruolo	Vittima
Cultura	Lavoro	Padre	Scelta	Voce
Cuore	Legge	Paese	Scena	Volta
Direttore	Lettera	Pagina	Scuola	Voto
Diritto	Letto	Parola	Secolo	Zona
